# Physiological predictors of hematological tier outcomes in canine platelet-rich plasma: a prospective observational study

**DOI:** 10.3389/fvets.2026.1904825

**Published:** 2026-07-30

**Authors:** Rocío Colomer-Selva, Asta Tvarijonaviciute, Lorena Franco-Martínez, Emma Martins, Beatriz Mena-Moros, María Descalzo-Navarro, Ángel Hernández-Guerra, Katy Satué

**Affiliations:** 1 Department of Animal Medicine and Surgery, Faculty of Veterinary Medicine, BIOMED SURGERY Group, CEU-Cardenal Herrera University, Valencia, Spain; 2 Interdisciplinary Laboratory of Clinical Analysis (Interlab-UMU), Regional Campus of International Excellence “Campus MareNostrum”, University of Murcia, Murcia, Spain; 3 Clinic for Internal Diseases, Faculty of Veterinary Medicine, University of Zagreb, Zagreb, Croatia

**Keywords:** body condition score, canine, hematological variability, platelet enrichment, platelet structural phenotype, platelet-rich plasma

## Abstract

Platelet-rich plasma (PRP) exhibits substantial inter-individual variability, yet the physiological factors influencing its composition remain poorly defined. Most studies evaluate PRP as a homogeneous product, potentially masking associations between donor-related characteristics and platelet parameters. This study aimed to determine how key physiological factors influence whole blood (WB) and PRP indices and to assess their relationship with platelet enrichment. Ninety-seven client-owned dogs were categorized according to sex, age, body condition score (BCS), fertility status, and activity level. PRP samples were stratified according to platelet enrichment (Q1 < 1.5×, Q2 1.5–2.5×, Q3 > 2.5×), which served as a descriptive framework for characterizing PRP heterogeneity. WB and PRP indices were analyzed using univariate methods and multivariable models, including linear regression to identify predictors of platelet enrichment and ordinal logistic regression as a complementary approach. Physiological factors were associated with variability in platelet morphological and compositional indices. Among the variables evaluated, BCS was the only physiological factor consistently associated with platelet enrichment across multivariable analyses. Higher BCS was associated with increased platelet enrichment and a higher probability of belonging to higher hematological enrichment tiers. Age was negatively associated with platelet enrichment in the multivariable linear regression model, but this association was not retained in the ordinal regression analysis. In contrast, sex, fertility status, and activity level showed no independent associations with platelet enrichment after adjustment for potential confounders. Correlation analyses revealed strong clustering among concentration-related indices [platelet count (PLT), plateletcrit (PCT), and large platelets (LARGEPLT)] and a consistent internal organization of platelet parameters within both WB and PRP. These findings indicate that platelet enrichment is primarily determined by preparation-related factors, whereas physiological factors contribute mainly to inter-individual variability in platelet structural characteristics. Among the physiological factors evaluated, BCS appeared to be the most relevant source of biological variability in platelet enrichment. Platelet enrichment and platelet structural phenotype therefore represent related but distinct dimensions of PRP characterization. Future studies incorporating functional and biochemical markers are needed to determine the biological significance of the structural variability described here.

## Graphical abstract

**Figure:**
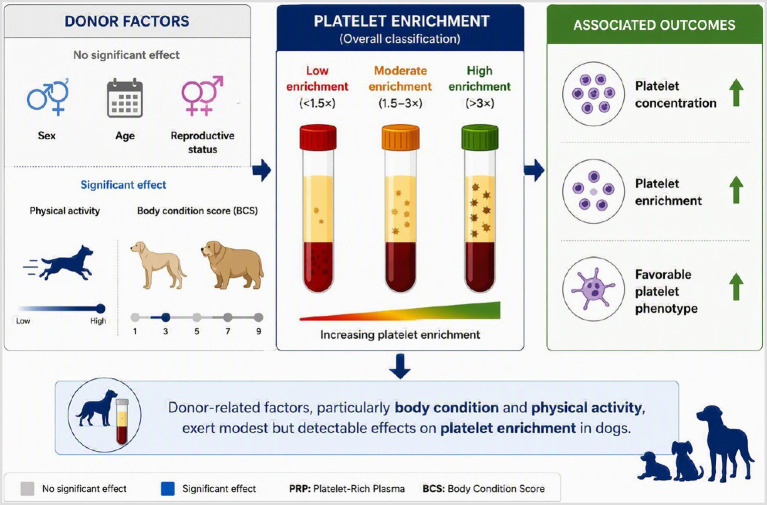

## 1 Introduction

Autologous platelet concentrates (APCs) are regenerative blood-derived products prepared from autologous blood and enriched in platelets and bioactive mediators involved in tissue healing and regeneration. Based on their leukocyte and fibrin content, APCs are generally classified into four categories: pure platelet-rich plasma (P-PRP), leukocyte- and platelet-rich plasma (L-PRP), pure platelet-rich fibrin (P-PRF), and leukocyte- and platelet-rich fibrin (L-PRF) (1, 2). Platelet-rich plasma (PRP), the most widely used APC, is a plasma fraction obtained by centrifugation of autologous blood that contains platelet concentrations above physiological whole-blood (WB) levels (3).

The intrinsic hematological profile of platelet concentrates—rather than platelet number alone—is increasingly recognized as a key determinant of the biological performance and therapeutic efficacy of PRP. In veterinary medicine, substantial interindividual variability in platelet characteristics complicates the standardization of PRP products, and the interpretation of hematologic measurements (4). This variability is influenced by physiological factors such as age, sex, body condition, physical activity and reproductive status, all of which can modulate platelet structure and composition. In the present study, PRP is characterized exclusively in hematological terms, focusing on platelet-related variables that describe its structural phenotype. This approach provides a pragmatic and clinically accessible framework to investigate variability in platelet profiles, while recognizing that it represents only one dimension of PRP characterization and does not capture functional or biochemical properties.

Sex is a relevant source of variation in PRP hematological characteristics. Human studies report sex-dependent differences in PRP growth factor content and platelet activation (5–8), largely driven by hormonal regulation. Sex-related hormonal status also influences leukocyte subpopulations and immune cell profiles in whole blood, changes that may persist after PRP preparation and contribute to variability in PRP composition. Comparable findings in horses (9, 10) and dogs (11) indicate that sex influences platelet morphology, composition, and related indices across species.

Reproductive physiology contributes additional variability to platelet biology. Human studies report sex and age dependent differences in PRP growth factor content (5, 6, 8), while veterinary evidence shows that reproductive status influences platelet morphology and growth factor release in horses (9, 10) and dogs (11).

Broader physiological literature further supports the role of endocrine and mitochondrial regulation in modulating platelet activation potential (12). However, the independent contribution of reproductive status to platelet enrichment remains uncertain, as most studies focus on functional markers rather than structural hematological indices.

Age is another well-established modulator of platelet biology. Healthy dogs show age-related differences in platelet indices (13), and similar shifts in PRP hematological parameters have been reported in horses (9, 14, 15) and humans (16). Comparative studies further indicate that age influences both platelet quantity and structural attributes relevant to PRP characterization (17, 18). However, the extent to which age independently affects platelet enrichment remains unclear, as most studies do not evaluate enrichment as a continuous outcome.

Body condition score (BCS) also shapes PRP hematological characteristics across species. In humans, obesity is associated with altered platelet morphology, heightened activation and proinflammatory phenotypes (19). Veterinary studies report comparable body mass related differences in platelet indices and PRP composition (11, 20), and comparative analyses identify BCS as a relevant modulator of PRP hematological variation (17). Given these findings, BCS represents a biologically plausible candidate to influence platelet enrichment, although its independent contribution has not been fully established in canine PRP.

Physical activity also modulates platelet biology. Exercise alters platelet energetics, mitochondrial metabolism and activation thresholds, producing measurable changes in PRP composition (21, 22). Short term exercise can modify platelet granule content and generate “exercise mobilized PRP” with distinct biological properties (23), and genetic studies further support a causal link between habitual activity and platelet morphology and function (24). Nevertheless, the extent to which activity level influences platelet enrichment—beyond its effects on platelet activation and granule dynamics—has not been systematically evaluated in dogs.

Even under standardized protocols, canine PRP exhibits substantial variability in platelet enrichment and hematology-based indices (25). This variability is clinically relevant because PRP composition involves multiple structural platelet characteristics, including platelet mass, morphology, granule content and heterogeneity (26). Contemporary guidelines emphasize that inadequate consideration of donor physiology is a major source of inconsistency in PRP research (27). Although platelet enrichment does not capture functional or biochemical dimensions of PRP, it remains a widely used structural descriptor in veterinary medicine. Therefore, enrichment-based stratification provides a practical and reproducible approach to reduce technical variability and compare PRP preparations according to their structural hematological potential. In this context, stratifying PRP by enrichment allows physiological influences on the platelet structural phenotype to be evaluated within comparable analytical levels.

Platelet-rich plasma hematological characteristics varies markedly among individuals, reflecting substantial variability in platelet concentration, morphology, mass and heterogeneity. This intrinsic heterogeneity can mask the true influence of donor physiology if PRP is treated as a homogeneous product. Most previous studies have evaluated physiological effects on PRP using unclassified preparations, an approach that overlooks this variability and may obscure meaningful associations between intrinsic traits and platelet phenotypic variability. Moreover, no study has simultaneously assessed how age, sex, body condition, reproductive status and activity level influence structural phenotype and the probability of generating PRP within defined hematological tiers based on platelet concentration (Q) (Q1 < 1.5×, Q2 1.5–2.5×, Q3 > 2.5×), used here as a descriptive stratification tool. In this context, stratifying PRP into predefined enrichment-based tiers allows physiological effects to be evaluated within comparable hematological levels, while recognizing that functional and biochemical dimensions of PRP characterization remain to be incorporated in future studies. This tiered approach provides a structured analytical framework to disentangle physiological influences on platelet structure from those affecting platelet concentration.

To address this gap, we stratified canine PRP into three hematological tiers based on platelet concentration and then evaluated how key physiological factors influence platelet indices and the distribution of samples across hematological tiers.

## 2 Materials and methods

### 2.1 Patient recruitment

This prospective clinical study was approved by the CEU Cardenal Herrera University Committee of Ethics in Animal Research and by the relevant competent authorities (code: 2024 VSC PEA 0146), in accordance with Spanish animal protection legislation (RD 53/2013) and the European Directive 2010/63/EU. A total of 97 client owned dogs (44 males and 53 females) representing 30 breeds and mixed breed backgrounds were included. The inclusion criteria comprised clinically healthy dogs presented to the CEU Veterinary Teaching Hospital (HCV CEU) between June 2024 and September 2025 for elective procedures, primarily ovariohysterectomy/orchiectomy and dental prophylaxis. Health status was confirmed by physical examination and routine preoperative evaluation. Dogs with no history or clinical evidence of systemic, hematological, or inflammatory disease and not receiving medications known to affect platelet function were considered eligible for inclusion. All dogs were enrolled after their owners received detailed information about the study and provided written informed consent.

Dogs were assigned to three age categories for analysis: 1–4 years (*n* = 47), 5–9 years (*n* = 26), and 9–15 years (*n* = 24). BCS was scored using the World Small Animal Veterinary Association (WSAVA) point BCS scale and categorized as underweight (BCS 1–3; *n* = 23), ideal weight (BCS 4–5; *n* = 50), or overweight (BCS 6–7; *n* = 24) (28). Reproductive status was recorded as fertile (intact; *n* = 70) or infertile (neutered; *n* = 27). Activity level was based on owner reported daily routines and categorized as low (*n* = 11; <30 min of walking/day with minimal spontaneous activity), medium (*n* = 50; 35–90 min/day with moderate spontaneous activity such as greeting behaviors or occasional play), or high (*n* = 36; >90 min/day with frequent spontaneous play, running, or continuous movement). Owners were advised that stress or anxiety-related behaviors could be perceived as high activity.

Exclusion criteria included dogs younger than 1 year of age; animals with systemic, inflammatory, infectious, hematological, or metabolic disorders; and dogs presenting hematological or biochemical abnormalities on routine preoperative screening. Animals receiving medications known to affect platelet number or function, including corticosteroids, non-steroidal anti-inflammatory drugs (NSAIDs), antiplatelet agents, or anticoagulants, were also excluded. Pregnant or lactating females were not eligible for inclusion. Additional exclusion criteria comprised recent strenuous exercise, which may induce transient alterations in platelet activation and hematological parameters and therefore confound the evaluation of habitual activity-related differences, marked stress or anxiety that could interfere with platelet activation, and preanalytical sample-related issues, such as hemolysis, clot formation, or insufficient blood volume for analysis. Dogs whose owners did not provide written informed consent were also excluded.

### 2.2 Blood collection

As part of the standard anesthetic premedication protocol required for their scheduled elective procedures, dogs were sedated with methadone (0.1 mg/kg intramuscularly) and dexmedetomidine (2 μg/kg). Once adequate sedation was achieved and prior to the administration of any induction agents or the commencement of surgery, all blood sampling was performed. First, an initial 0.5 mL blood sample was collected from the jugular vein using a 21gauge butterfly needle into a K3EDTA tube (BD Vacutainer; Becton Dickinson) for whole blood analysis. Hematology results were reviewed immediately, and only dogs showing no hematological abnormalities proceeded with the subsequent PRGF® Endoret® preparation.

Following confirmation of normal hematology and within the same pre-surgical window, 9 mL of blood were collected from the same vein under sterile conditions into a vacutainer containing 0.4 mL of sodium citrate anticoagulant (Blood Collecting Tubes®, BTI Biotechnology Institute, Álava, Spain) for PRP preparation and extraction, and subsequent platelet indices analysis. All samples were handled gently to avoid platelet activation prior to processing.

### 2.3 PRP preparation

PRP was obtained following the PRGF Endoret® methodology (BTI Biotechnology Institute). Citrated whole blood was centrifuged at room temperature using a validated protocol of 460 g for 8 min (BTI Vet System IV). Centrifugation yielded three layers: red blood cells, the buffy coat, and plasma. According to the manufacturer’s specifications, the upper 60% of the plasma was considered the platelet poor plasma (PPP) fraction, whereas the lower 40%, located immediately above the buffy coat, corresponded to the PRP fraction. The PRP layer was aspirated carefully under aseptic conditions and always by the same researcher to minimize variability. The collected PRP was then aliquoted into Eppendorf tubes for subsequent platelet indices analysis.

### 2.4 Whole blood and PRP analysis

Platelet indices were measured in WB and PRP using the ADVIA 2120i hematology analyzer (Siemens Healthcare Diagnostics Inc.). For interpretative clarity, variables were grouped into two categories: quantitative platelet parameters, and morphological, mass related or heterogeneity indices. Quantitative parameters included platelet count (PLT; 10^3^/μL), plateletcrit (PCT; %), mean platelet volume (MPV; fL), large platelets (LARGEPLT; 10^3^/μL) and platelet clump count (AGREG). Morphological and composition related indices included platelet distribution width (PDW; %), mean platelet component (MPC; g/dL), mean platelet mass (MPM; pg), platelet dry mass distribution width (PMDW) and platelet component distribution width (PCDW; g/dL). MPC reflects the internal refractive index or protein density of platelets, PCDW represents the dispersion of MPC values, and MPM corresponds to the estimated mean platelet mass; these three indices are specific to the ADVIA analytical platform. Manual microscopic assessment of platelet activation, although informative, is subject to observer variability and is not feasible for large clinical cohorts. Therefore, automated indices were used as the primary structural markers of platelet phenotype in this study, consistent with the study’s focus on hematological characterization rather than functional assessment.

### 2.5 PRP hematological tier classification

To evaluate the influence of sex, age, BCS, fertility and activity level on PRP characteristics, samples were classified into three hematological tiers according to their platelet enrichment relative to WB: Q1 (low hematological tier, <1.5-fold increase), Q2 (intermediate hematological tier, 1.5–2.5-fold increase) and Q3 (higher enrichment tier, >2.5-fold increase).

This classification is based on platelet enrichment thresholds used as a descriptive framework to stratify samples according to their relative increase in platelet concentration. A ≥ 1.5-fold increase in platelet concentration was selected as a practical lower threshold to distinguish samples from those with minimal or no enrichment, as comparative analyses of commercial canine PRP systems demonstrate that some preparations fail to raise platelet counts above WB levels (29). The upper tier (Q3, >2.5-fold enrichment) was defined to differentiate samples with higher levels of platelet concentration, as enrichment magnitude is commonly used to describe variability among PRP preparations (1). In addition, veterinary PRP reporting guidelines recommend stratifying samples according to enrichment level to improve comparability and consistency across studies (30). Comparative studies further demonstrate that a wide range of platelet concentrations can be technically achieved in canine PRP preparations (29). This tiered classification provides a reproducible framework for describing variability and evaluating physiological influences on PRP hematological characteristics. Importantly, these hematological tiers should be interpreted exclusively as a structural stratification framework and do not imply differences in biological quality, functional activity, or therapeutic efficacy.

### 2.6 Statistical analysis

Data from WB and PRP were summarized descriptively. Continuous variables were expressed as median and interquartile range (IQR), and categorical variables as frequencies and percentages. The distribution of continuous variables was assessed using the Shapiro–Wilk test and was found to deviate from normality.

Paired WB and PRP measurements were compared using the Wilcoxon signed-rank test. Comparisons between two-category physiological variables (sex and fertility status) were performed using the Mann–Whitney U test. Comparisons among variables with more than two categories (age group, BCS, and activity level) were performed using the Kruskal–Wallis test followed by Dunn’s *post hoc* test when appropriate.

To reduce the risk of false-positive findings arising from multiple subgroup comparisons, *p*-values from exploratory univariate analyses were adjusted using the Benjamini–Hochberg false discovery rate (FDR) procedure. Multivariable regression models were considered the primary inferential analyses and are therefore reported using unadjusted *p*-values.

Given that platelet enrichment represents the primary quantitative outcome of PRP preparation, the main inferential analysis was performed using a multivariable linear regression model. The enrichment factor (F_CONCENTR) was included as the dependent variable, whereas sex, age group, BCS, fertility status, and activity level were included as explanatory variables. Regression coefficients (*β*), 95% confidence intervals (95% CI), and exact *p*-values were calculated. Model performance was assessed using the coefficient of determination (*R*^2^) and adjusted R^2^. The assumptions of the linear regression model, including homoscedasticity, normality of residuals, and multicollinearity, were evaluated by visual inspection of residual plots and assessment of variance inflation factors (VIF).

As a complementary analysis, hematological tiers were evaluated using an ordinal logistic regression model (proportional odds model). Hematological tier (Q1 < Q2 < Q3) was included as the dependent variable, whereas sex, age group, BCS, fertility status, and activity level were included as explanatory variables. Results are presented as odds ratios (OR) with 95% confidence intervals and exact *p*-values. The proportional odds assumption was assessed using the Brant test.

Finally, correlations among platelet indices within WB and PRP, as well as between both matrices, were evaluated using Pearson’s or Spearman’s correlation coefficients according to distributional assumptions. Statistical significance was set at *p* < 0.05. Statistical analyses were performed using Statistica 16.0 (TIBCO Software Inc., Palo Alto, CA, USA) running on Microsoft Windows 11.

## 3 Results

### 3.1 Descriptive analyses and subgroup comparisons

Descriptive statistics for WB and PRP variables are presented in Table 1.

**Table 1:** Descriptive statistics (median and 25th–75th percentiles) of whole blood (WB) and platelet-rich plasma (PRP) variables for the full dataset, presented without adjustment for physiological variables.

Parameter	WB	PRP
PLT (10^3^/mL)	294.6 (232.2–346.2)	568.7 (325.5–772.2)
PCT (%)	0.32 (0.24–0.37)	0.77 (0.38–1.07)
MPV (fL)	11.5 (10.0–11.6)	13.2 (10.7–15.3)
LARGEPLT (10^3^/mL)	21.5 (10.2–24.0)	101.6 (22–154)
MPC (g/dL)	23.4 (22.8–24.6)	20.6 (17.8–23.4)
PDW (%)	63.9 (58.0–69.2)	57.7 (56.0–60.2)
PCDW (g/dL)	5.87 (4.80–6.70)	7.65 (7.10–8.70)
MPM (pg)	2.31 (2.11–2.45)	2.20 (2.03–2.32)
PMDW (pg)	0.91 (0.84–0.97)	0.77 (0.68–0.83)
AGREG (n)	187.7 (34.0–242.0)	39.9 (13.2–40.5)

#### 3.1.1 Effect of sex

Sex-related differences were observed in both WB and PRP platelet indices (Supplementary Table 1). In WB, differences primarily involved PLT, PCT, MPV, MPC, and PMDW, whereas in PRP they mainly involved MPV, LARGEPLT, PDW, PCDW, PMDW, MPC, and AGREG. Differences were most evident at intermediate and higher enrichment levels, with females generally exhibiting lower MPV, LARGEPLT, MPM, and PMDW values than males; similarly, WB MPC was higher in males than females at the intermediate enrichment level.

#### 3.1.2 Effect of age

Age-related differences primarily involved platelet morphological parameters (Supplementary Table 2). Differences were mainly observed in PRP, where MPV and LARGEPLT tended to increase with platelet enrichment, whereas younger dogs showed higher MPC values.

#### 3.1.3 Effect of body condition score (BCS)

BCS-related differences were observed in both WB and PRP platelet indices (Supplementary Table 3). In WB, differences primarily involved MPC, whereas in PRP the main differences involved AGREG, MPV, and MPC, particularly at lower and higher enrichment levels.

#### 3.1.4 Effect of fertility status

Fertility-related differences were observed in both WB and PRP platelet indices (Supplementary Table 4). In WB, differences primarily involved PLT and MPC. In PRP, differences involved MPV, LARGEPLT, PCDW, AGREG, and MPC, particularly at intermediate and higher enrichment levels. Across hematological tiers, fertile animals generally exhibited higher MPC values, whereas differences in other platelet indices were inconsistent.

#### 3.1.5 Effect of physical activity

Activity-related differences primarily affected PRP platelet indices (Supplementary Table 5). Differences involved PLT, PCT, LARGEPLT, MPV, PCDW, MPM, AGREG, and PMDW across enrichment categories and activity levels. Across hematological tiers, low-activity dogs generally exhibited higher MPV, MPM, and PMDW values than more active animals.

### 3.2 Multivariable analysis

#### 3.2.1 Linear regression model of platelet enrichment

A multivariable linear regression model was fitted using platelet enrichment (F_CONCENTR) as the dependent variable and sex, age group, BCS, fertility status, and activity level as explanatory variables. Diagnostic evaluation indicated no major violations of model assumptions, including homoscedasticity, normality of residuals, and multicollinearity.

**Figure 1:**
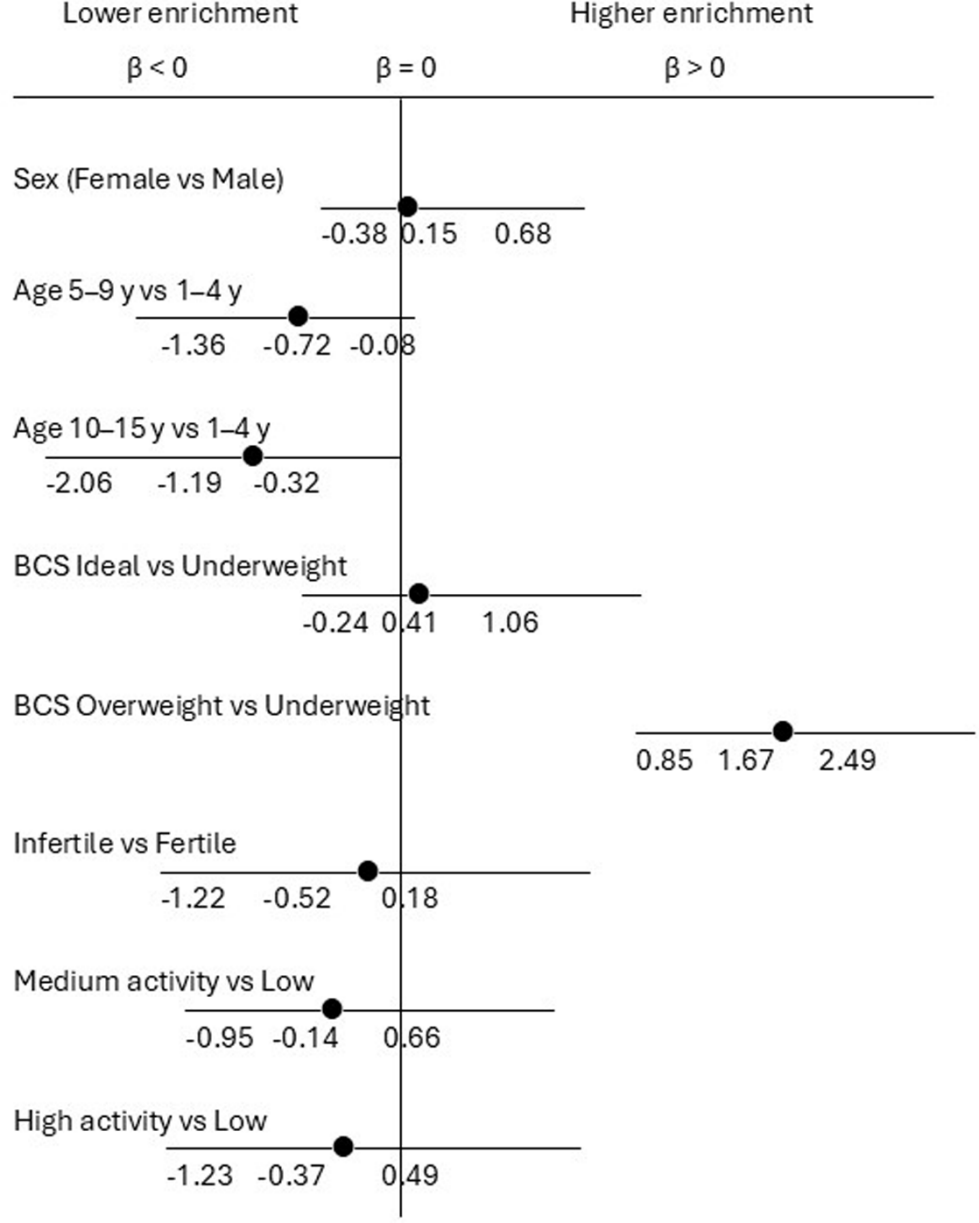
Forest plot of the multivariable linear regression model evaluating physiological predictors of platelet enrichment (F_CONCENTR). Points represent regression coefficients (β) and horizontal bars represent 95% confidence intervals (95% CI). The vertical line indicates no association (β = 0). Positive coefficients indicate higher platelet enrichment relative to the reference category, whereas negative coefficients indicate lower platelet enrichment.

The multivariable linear regression model explained 17.4% of the variability in platelet enrichment (*R*^2^ = 0.174; adjusted *R*^2^ = 0.118) (Table 2 and Figure 1).

**Table 2:** Multivariable linear regression model evaluating the association between physiological variables and platelet enrichment (F_CONCENTR).

Predictor	*β*	95% CI	*p*-value
Intercept	2.124	1.17 to 3.07	<0.001
Sex (Female vs. Male)	0.151	−0.38 to 0.68	0.574
Age 5–9 y vs. 1–4 y	−0.720	−1.36 to −0.08	0.028
Age 10–15 y vs. 1–4 y	−1.187	−2.05 to −0.31	0.008
BCS Ideal vs. Underweight	0.412	−0.24 to 1.06	0.214
BCS Overweight vs. Underweight	1.674	0.85 to 2.49	<0.001
Infertile vs. Fertile	−0.517	−1.21 to 0.18	0.146
Medium activity vs. Low activity	−0.143	−0.95 to 0.66	0.726
High activity vs. Low activity	−0.367	−1.23 to 0.49	0.400

Regression coefficients (β), 95% confidence intervals (95% CI), and exact *p*-values are shown for each predictor included in the model. Positive *β* values indicate higher platelet enrichment relative to the reference category, whereas negative *β* values indicate lower platelet enrichment. Model performance was *R*^2^ = 0.174 and adjusted *R*^2^ = 0.118.

BCS was independently associated with platelet enrichment. Compared with underweight dogs, dogs in the highest BCS category showed an increase of 1.67 units in platelet enrichment (*β* = 1.67; 95% CI: 0.85–2.49; *p* < 0.001), whereas no significant differences were detected between the intermediate and low BCS categories (*β* = 0.41; 95% CI: −0.24 to 1.06; *p* = 0.214).

Age was independently associated with platelet enrichment. Compared with the youngest age group, enrichment decreased by 0.72 units in dogs aged 5–9 years (*β* = −0.72; 95% CI: −1.36 to −0.08; *p* = 0.028) and by 1.19 units in dogs aged 10–15 years (*β* = −1.19; 95% CI: −2.06 to −0.32; *p* = 0.008).

Sex, fertility status, and activity level were not independently associated with platelet enrichment after multivariable adjustment (all *p* > 0.05).

#### 3.2.2 Ordinal logistic regression

**Figure 2:**
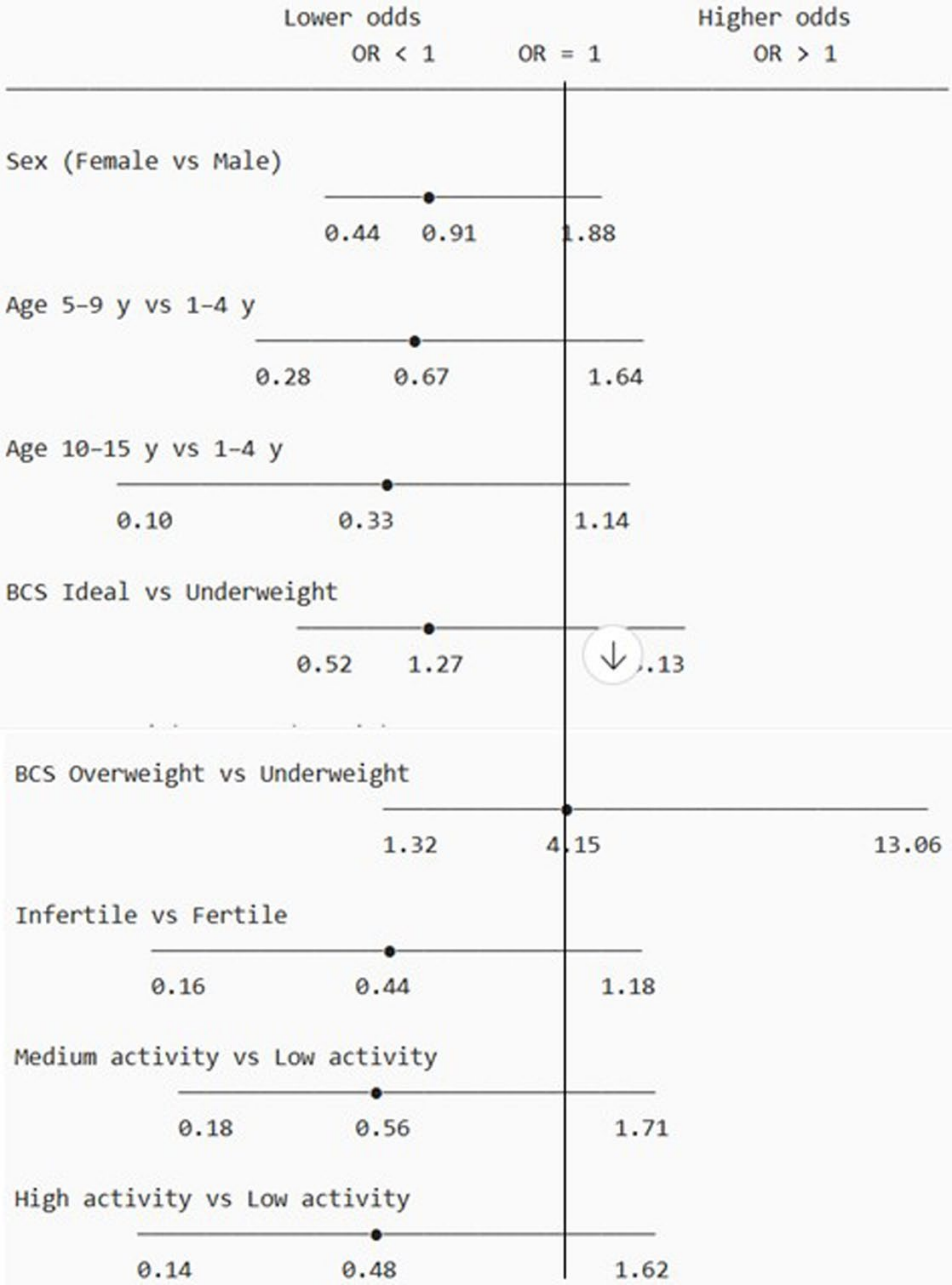
Forest plot of the ordinal logistic regression model evaluating physiological predictors of hematological tier allocation (Q1 < Q2 < Q3). Odds ratios (OR) and corresponding 95% confidence intervals (95% CI) are shown for all predictors included in the model. Points represent odds ratios and horizontal bars represent 95% confidence intervals. The vertical line indicates no association (OR = 1). Odds ratios greater than 1 indicate increased odds of belonging to a higher hematological enrichment tier, whereas odds ratios lower than 1 indicate decreased odds relative to the reference category.

As a secondary analysis, an ordinal logistic regression model was performed using hematological tier (Q1 < Q2 < Q3) as the dependent variable (Table 3 and Figure 2).

**Table 3:** Ordinal logistic regression model evaluating physiological predictors of hematological tier allocation (Q1 < Q2 < Q3).

Predictor	OR	95% CI	*p*-value
Sex (Female vs. Male)	0.91	0.44–1.88	0.806
Age 5–9 y vs. 1–4 y	0.67	0.28–1.64	0.384
Age 10–15 y vs. 1–4 y	0.33	0.10–1.14	0.079
BCS ideal vs. Underweight	1.27	0.52–3.13	0.600
BCS Overweight vs. Underweight	4.15	1.32–13.06	0.015
Infertile vs. Fertile	0.44	0.16–1.18	0.103
Medium activity vs. Low activity	0.56	0.18–1.71	0.307
High activity vs. Low activity	0.48	0.14–1.62	0.237

Odds ratios (OR), 95% confidence intervals (95% CI), and exact p-values are presented for all predictors included in the model. Odds ratios greater than 1 indicate increased odds of belonging to a higher hematological enrichment tier, whereas odds ratios lower than 1 indicate decreased odds relative to the reference category. The proportional odds assumption was satisfied, as indicated by the Brant test (Table 4).

BCS was the only variable independently associated with hematological tier allocation in the ordinal regression model. Dogs in the highest BCS category were significantly more likely to belong to higher enrichment tiers than dogs in the lowest BCS category (OR = 4.15; 95% CI: 1.32–13.06; *p* = 0.015).

No significant associations were identified for sex, age, fertility status, or activity level, although the oldest age group showed a non-significant tendency toward lower odds of belonging to higher enrichment tiers (OR = 0.33; 95% CI: 0.10–1.14; *p* = 0.079).

The proportional odds assumption was evaluated using the Brant test (Table 4). No significant violations were detected for any individual predictor, and the global test indicated no evidence of violation of the proportional odds assumption (χ^2^ = 2.11, df = 8, *p* = 0.978).

**Table 4:** Assessment of the proportional odds assumption for the ordinal logistic regression model using the Brant test.

Predictor	χ^2^	*p*-value
Sex	0.012	0.914
Age (5–9 y vs. 1–4 y)	0.052	0.820
Age (10–15 y vs. 1–4 y)	0.321	0.571
BCS (Ideal vs. Underweight)	0.087	0.768
BCS (Overweight vs. Underweight)	0.767	0.382
Fertility status	0.054	0.816
Medium activity vs. Low activity	0.026	0.871
High activity vs. Low activity	0.788	0.375
Global Brant test (df = 08)	**2.11**	**0.978**

The Brant test was used to assess the proportional odds assumption. Non-significant p-values indicate no evidence of violation of the proportional odds assumption for individual predictors or for the overall model. Bold values denote significant correlations (*P* < 0.05).

**Figure 3:**
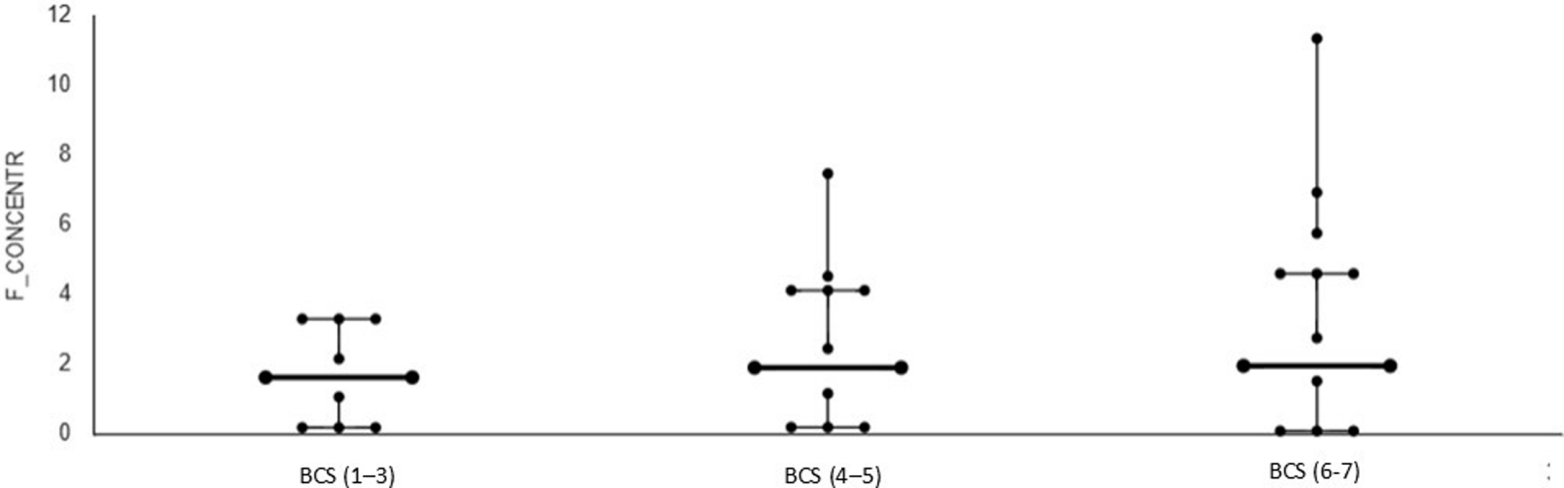
Distribution of platelet enrichment (F_CONCENTR) according to body condition score (BCS). Boxplots display the median (central line), interquartile range (IQR; box), whiskers, and individual observations across BCS categories.

The distribution of F_CONCENTR across BCS categories is presented in Figure 3.

### 3.3 Correlation analysis

Statistically significant correlations were identified among platelet indices within WB, within PRP, and between both matrices (Table 5). In WB, the strongest correlations were observed between PLT and PCT, and between MPV and LARGEPLT. In PRP, the highest correlations involved PLT, PCT, LARGEPLT, MPM, and PMDW. Significant correlations were also detected between WB and PRP measurements, particularly for MPM and PLT. A weak negative correlation was observed between MPVWB and PLTPRP. All reported correlations were statistically significant (*p* < 0.05).

**Table 5:** Summary of significant correlations (*r*) between whole blood (WB) and platelet-rich plasma (PRP) indices.

Spearman’s correlations					
Parameter pair (WB)	*r*	Parameter pair (PRP)	*r*	Parameter pair (WB ↔ PRP)	*r*
PLT–PCT	0.81	PLT – PCT	0.95	MPM_WB – MPM_PRP	0.66
MPV–LARGEPLT	0.80	PCT – LARGEPLT	0.92	PLT_WB – PLT_PRP	0.63
MPV–PMDW	0.78	MPM – PMDW	0.89	PLT_WB – PCT_PRP	0.59
MPV–MPM	0.78	PLT – LARGEPLT	0.78	MPV _WB – PLT_PRP	−0.26
LARGEPLT–PMDW	0.62	MPV – MPC	0.77		
LARGEPLT–PCDW	0.60	LARGEPLT – PCDW	0.67		
LARGEPLT–PDW	0.59	LARGEPLT – MPC	0.65		
		MPV – PCDW	0.65		

Correlations are grouped by matrix (PRP, WB and WB↔PRP). All associations were statistically significant (*p* < 0.05).

## 4 Discussion

The present study investigated the contribution of physiological factors to platelet enrichment and platelet-related structural variability in canine PRP. The principal finding was that BCS emerged as the most consistent physiological predictor of platelet enrichment. Higher BCS was associated with increased platelet enrichment in both the multivariable linear and ordinal regression models, whereas sex, fertility status, and activity level were not independently associated with enrichment after adjustment for potential confounders. In addition, age was negatively associated with platelet enrichment in the linear regression model, suggesting that both BCS and age may contribute to inter-individual variability in PRP composition.

A second important finding was that platelet enrichment and platelet structural phenotype behaved as related but distinct dimensions. Although platelet concentration-related variables, including PLT, PCT, and LARGEPLT, increased consistently across enrichment levels, several morphological and compositional indices showed weaker or non-linear relationships. These results indicate that platelet enrichment alone does not fully capture the complexity of the platelet phenotype and support the use of a broader hematological characterization of PRP, consistent with previous studies emphasizing the multifactorial nature of PRP composition and biological variability (9, 10, 21).

The WB and PRP profiles observed in this study are consistent with current evidence showing that concentration processes increase platelet count, PCT, and the proportion of LARGEPLT, while modifying platelet size and dispersion indices. These changes, consistently reported in recent canine studies, reflect the redistribution of platelet subpopulations during PRP preparation (17, 31). The strong internal clustering identified in PRP, particularly the close association among PLT, PCT, and LARGEPLT, supports the interpretation that platelet concentration-related parameters behave as a coordinated structural module. A similar organization in WB suggests that baseline platelet characteristics contribute to this pattern. The moderate correspondence between WB and PRP indices is also expected, as initial platelet count and morphology influence platelet recovery during centrifugation (32). Together, these findings reinforce that platelet enrichment is primarily associated with preparation-related factors, whereas physiological variables are mainly associated with variability in platelet structural characteristics.

A notable strength of this study was the stratification of samples according to platelet enrichment, which allowed physiological influences to be evaluated within comparable enrichment levels rather than in heterogeneous PRP populations. This approach reduces the potential masking effect of variability in platelet recovery and provides a structured framework for describing PRP heterogeneity. Furthermore, the use of multivariable models allowed the identification of independent predictors of enrichment, thereby improving the interpretation of physiological influences on PRP characteristics.

### 4.1 Effect of age

Age was independently associated with platelet enrichment in the multivariable linear regression model, with older dogs showing lower enrichment values than younger animals. This finding suggests that age-related changes in platelet production, morphology, or turnover may influence platelet recovery during PRP preparation. However, age was not significantly associated with hematological tier allocation in the ordinal regression model, indicating that its effect was less robust than that observed for BCS.

Age-related changes in platelet physiology have been described in both human and veterinary medicine and may involve alterations in platelet turnover, megakaryocyte activity, and platelet morphology (14–16). These mechanisms could contribute to the lower enrichment values observed in older dogs, although the observed effect should be interpreted cautiously. The descriptive analyses further showed higher MPV and LARGEPLT values in older dogs and higher MPC values in younger animals. Similar age-associated variations in platelet morphology have previously been reported in both veterinary and human studies, whereas effects on platelet concentration have generally been inconsistent (14–16).

The discrepancy between the linear and ordinal models suggests that age may influence the magnitude of enrichment as a continuous trait without necessarily determining allocation to discrete enrichment categories. Together, these findings suggest that age contributes to variability in platelet structural characteristics, while its association with PRP enrichment appears less consistent than that observed for body condition score.

### 4.2 Effect of BCS

Body condition score emerged as the most consistent physiological predictor of platelet enrichment. Higher BCS was associated with increased platelet enrichment in the multivariable linear regression model, and overweight dogs were significantly more likely to belong to higher hematological enrichment tiers in the ordinal regression analysis. This finding indicates that body condition contributes to inter-individual variability in PRP enrichment independently of other physiological characteristics.

The biological mechanisms underlying this association remain unclear. Body condition has been associated with low-grade systemic inflammation, metabolic alterations, and changes in platelet physiology, inflammatory mediators, and hemostatic regulation in both humans and dogs (4, 17, 33). These factors may influence platelet recovery during centrifugation by altering platelet size, density, or activation state. Although the present study did not evaluate inflammatory biomarkers or platelet function, the consistent association between BCS and platelet enrichment across both multivariable models suggests that adiposity may represent a relevant source of biological variability in PRP preparation.

Descriptive analyses also identified differences in several platelet morphological and compositional indices among BCS categories. However, these differences were less consistent than the associations observed for platelet enrichment and primarily affected structural characteristics rather than platelet concentration. Previous studies similarly report inconsistent associations between body condition and platelet counts, whereas associations with platelet size and related indices have been described more frequently (11, 20, 33). Collectively, these findings suggest that BCS is associated with both platelet enrichment and aspects of platelet phenotype.

Adipose tissue is increasingly recognized as an active endocrine and inflammatory organ that contributes to systemic metabolic and immune regulation (17, 33). Consequently, body condition may influence hematological and platelet-related variables through mechanisms that are not directly reflected by platelet count alone. This perspective is consistent with the observation that BCS was associated with enrichment despite the absence of clear differences in several conventional platelet concentration-related indices, suggesting that factors beyond platelet count alone may contribute to variability in platelet recovery.

The association between BCS and platelet enrichment may also be partially explained by differences in platelet morphology. Larger platelets are generally considered more metabolically active and may behave differently during concentration procedures. The descriptive differences observed in MPV and LARGEPLT across BCS categories therefore support the possibility that platelet structural characteristics contribute to the enrichment process itself.

From a practical perspective, these findings suggest that donor characteristics should not be considered completely negligible when interpreting PRP composition. Although preparation methodology remains the primary determinant of platelet recovery, body condition may represent an additional source of biological variability among individuals.

### 4.3 Effect of sex, fertility status, and physical activity

Sex, fertility status, and activity level were not independently associated with platelet enrichment in the multivariable analyses. Although some differences in platelet morphological and compositional indices were observed in the descriptive subgroup analyses, these findings were not consistently observed across enrichment levels and were not retained after multivariable adjustment.

For sex, the observed differences mainly involved platelet morphology, with males tending to exhibit higher MPV and LARGEPLT values at some enrichment levels. Similar findings have been reported previously, suggesting that sex-related influences are more frequently observed in platelet phenotype than in platelet enrichment itself (5, 6, 8, 34, 35).

Likewise, fertility status was not independently associated with platelet enrichment after multivariable adjustment. Although previous studies have reported associations between reproductive status and platelet morphology or growth factor release in veterinary species (9–11), these associations were not observed in the multivariable analyses performed in the present study.

Likewise, habitual physical activity showed little influence on platelet enrichment. Although low-activity dogs displayed differences in selected morphological indices, these effects were not consistently observed across enrichment levels and were not retained in the multivariable models. This observation is consistent with previous studies indicating that long-term activity patterns exert relatively minor effects on platelet characteristics compared with the transient changes associated with acute exercise (21–23). Together, these findings suggest that physical activity may primarily affect platelet function and activation state rather than the efficiency of platelet recovery during PRP preparation (21–24).

Overall, these findings suggest that sex, fertility status, and activity level may contribute to variability in platelet structural characteristics. However, none of these variables showed independent associations with platelet enrichment after multivariable adjustment.

The present study has several limitations that should be acknowledged. Although previous studies have suggested that platelet structural characteristics may be associated with growth factor release and functional properties (5, 8, 12, 19), such relationships cannot be assessed in the present work. As no functional assays or biochemical markers of activation were included, the findings reported here should be interpreted strictly as a characterization of structural variability. The relationship between platelet structure and functional output therefore remains an important area for future investigation. Future studies incorporating functional, biochemical, and activation-related markers will be necessary to determine how the structural phenotype described here translates into biological performance and therapeutic relevance.

Finally, although automated indices derived from the ADVIA 2120i provide standardized and reproducible information on platelet morphology, they do not replace direct morphological assessment by microscopy. Future studies integrating ultrastructural or imaging-based evaluation would complement the automated metrics used here. Such approaches would also help clarify how structural platelet features relate to activation dynamics and functional output, which could not be assessed in the present study.

In addition, the study was based on a single PRP preparation protocol. Therefore, the observed associations should be interpreted within the context of the methodology employed and may not be directly extrapolated to other PRP preparation systems or commercially available protocols.

Furthermore, the cross-sectional design of the study does not allow causal relationships between physiological variables and platelet enrichment to be established. Longitudinal studies would be useful to determine whether changes in factors such as BCS or age-related physiological status led to corresponding changes in PRP characteristics over time. Finally, although the sample size was relatively large for a veterinary PRP study, the distribution of animals across some physiological categories was not completely balanced. Therefore, subtle effects associated with specific subgroups cannot be entirely excluded.

Overall, the stratification applied in this study provides a structured framework for describing variability in platelet-related parameters while accounting for the heterogeneity inherent to PRP preparations. The results indicate that platelet enrichment is largely determined by the preparation process itself, although body condition score and, to a lesser extent, age may contribute to inter-individual variability. Nevertheless, the present framework should be interpreted as a description of structural hematological variability rather than as an indicator of biological quality, functional activity, or clinical efficacy. The present findings therefore characterize structural PRP phenotypes but do not imply functional equivalence or therapeutic comparability among enrichment tiers.

## 5 Conclusion

Among the physiological factors evaluated, BCS emerged as the physiological variable most consistently associated with platelet enrichment in canine PRP, whereas age showed an association in the linear regression model but not in the ordinal analysis. In contrast, sex, fertility status, and activity level were not independently associated with platelet enrichment.

Although physiological factors contributed to variability in platelet-related parameters, platelet enrichment was largely associated with preparation-related factors. Donor physiology appeared to influence mainly platelet morphological and compositional characteristics, whereas independent associations with platelet enrichment were identified only for body condition score and age. The enrichment-based stratification applied in this study proved useful for characterizing PRP heterogeneity and identifying physiological influences that might otherwise remain obscured in heterogeneous PRP populations.

These findings support the interpretation that platelet enrichment and platelet structural phenotype represent related but distinct dimensions of PRP characterization, each reflecting different aspects of biological variability. Future studies incorporating functional and biochemical markers will be necessary to determine the biological significance and therapeutic relevance of the structural variability described here.

## Data Availability

All data generated or analyzed during this study are available upon request from the corresponding authors. Requests to access the datasets should be directed to rocio.colomer@uchceu.es.
