# Supplementary material for: Physiological predictors of hematological tier outcomes in canine platelet-rich plasma: a prospective observational study
Source: Front Vet Sci. 2026 Jul 30;13:1904825. doi: 10.3389/fvets.2026.1904825 (PMC13470666; doi:10.3389/fvets.2026.1904825)
Supplement: Supplementary file 1 [file Table_1.DOC]

**Supplementary Table 1**. Platelet variables in whole blood (WB) and platelet-rich plasma (PRP) according to sex and hematological enrichment tier. Values are presented as median (25th–75th percentile). Mann–Whitney U and Kruskal–Wallis/Dunn tests were used, with Benjamini–Hochberg adjustment for multiple comparisons. Different superscript letters indicate significant differences among enrichment tiers within sex, and asterisks indicate significant differences between sexes within the same tier (adjusted p < 0.05).

Abbreviations: PLT, platelet count; PCT, plateletcrit; MPV, mean platelet volume; LARGEPLT, large platelets; MPC, mean platelet protein content; PDW, platelet distribution width; PCDW, platelet protein distribution width; MPM, mean platelet mass; PMDW, platelet mass distribution width; AGREG, platelet aggregates.

|  |  | **WB** | | | | | |  | **PRP** | | | | | | |
| --- | --- | --- | --- | --- | --- | --- | --- | --- | --- | --- | --- | --- | --- | --- | --- |
|  | **Sex** | **N** | **Q1 (< 1.5)** | **N** | **Q2 (1.5-2.5)** | **N** | **Q3 (> 2.5)** | **p-value** | **N** | **Q1 (< 1.5)** | **N** | **Q2 (1.5-2.5)** | **N** | **Q3 (> 2.5)** | **p-value** |
| **PLT (10³/mL)** | F | 24 | 264.5 (210.5–318.5)^a^ | 30 | 333.7 (270.5–396.9)^b^ | 19 | 289.0 (207.0–370.9)^a^ | **0.002** | 24 | 252.9 (158.7–347.2)^a^ | 30 | 662.5 (513.4–811.6)^b^ | 19 | 891.6 (676.4–1106.9)^c^ | **<0.001** |
|  | M | 20 | 278.1 (195.9–360.3) | 21 | 315.4 (259.8–370.9) | 12 | 261.5 (183.9–339.1) | ns | 20 | 248.2 (106.6–389.7)^a^ | 21 | 641.9 (527.4–756.5)^b^ | 12 | 874.8 (587.7–1161.8)^c^ | **<0.001** |
| **PCT (%)** | F | 24 | 0.3 (0.2–0.4)^a^ | 30 | 0.4 (0.3–0.5)^b^ | 19 | 0.3 (0.2–0.4)^a^ | **0.002** | 24 | 0.3 (0.2–0.4)^a^ | 30 | 0.9 (0.7–1.1)^b^ | 19 | 1.25 (0.9–1.6)^c^ | **<0.001** |
|  | M | 20 | 0.3 (0.2–0.4) | 21 | 0.3(0.3–0.4) | 12 | 0.3 (0.2–0.4) | ns | 20 | 0.3 (0.1–0.5)^a^ | 21 | 0.9 (0.7–1.1)^b^ | 12 | 1.4 (0.9–1.8)^c^ | **<0.001** |
| **MPV (fL)** | F | 24 | 11.7 (9.5–14.1) | 30 | 11.0 (9.6–12.4) | 19 | 10.6 (9.8–11.3) | ns | 24 | 11.6 (9.9–13.2)^a^ | 30 | 13.6 (11.7–15.5)^b^ | 19 | 13.6 (12.0–15.1)^b^ | **0.0028** |
|  | M | 20 | 12.7 (9.6–15.8)^a^ | 21 | 10.4 (9.2–11.6)^a^ | 12 | 14.6 (9.2–19.3)^b^ | **0.002** | 20 | 11.9 (9.7–14.1)^a^ | 21 | 13.5 (11.9–15.0)^a^ | 12 | 16.0 (15.1–16.9)^b^ | **0.0068** |
| **LARGEPLT (10³/mL)** | F | 24 | 24.9 (1.9–47.8)^a^ | 30 | 21.8 (5.3–38.3)a | 19 | 14.8 (8.9–20.8)^b^ | **0.033** | 24 | 28.5 (9.1–47.9)^a^ | 30 | 113.2 (63.5–162.9)^b^ | 19 | 157.1 (89.8–224.3)^c^ | **<0.001** |
|  | M | 20 | 26.6 (12.3–40.9) | 21 | 17.2 (6.3–28.1) | 12 | 23.9 (15.3–32.6) | ns | 20 | 38.3 (3.2–73.3)^a^ | 21 | 110.2 (67.2–153.1)^b^ | 12 | 220.5 (150.9–290.1)^c^ | **<0.001** |
| **MPC (g/dL)** | F | 24 | 23.5 (22.3–24.7) | 30 | 23.7 (22.8–24.6) | 19 | 23.3 (22.6–24.1) | ns | 24 | 22.2 (19.9–24.4) | 30 | 20.4 (18.3–22.5) | 19 | 20.2 (18.3–22.2) | **0.004** |
|  | M | 20 | 22.4 (20.9–23.9)^a^ | 21 | 24.4 (23.6–25.3)^b^ | 12 | 22.5 (19.95–25.1)^a^ | **0.004** | 20 | 21.4 (18.9–23.8) | 21 | 20.3 (18.6–22.1) | 12 | 18.5 (17.1–19.9) | ns |
| **PDW (%)** | F | 24 | 64.2 (58.9–69.6) | 30 | 63.3 (57.7–68.9) | 19 | 62.5 (57.2–67.9) | ns | 24 | 58.2 (50.4–66.1) | 30 | 57.2 (53.8–60.7) | 19 | 57.9 (54.5–61.3) | ns |
|  | M | 20 | 65.2 (57.7–72.7) | 21 | 62.9 (55.6–70.3) | 12 | 66.3 (59.2–73.3 | ns | 20 | 55.9 (50.0–61.9)^a^ | 21 | 57.9 (55.9–59.9)^a^ | 12 | 60.7 (58.9–62.4)^b^ | **0.002** |
| **PCDW (g/dL)** | F | 24 | 5.7 (4.9–6.6) | 30 | 5.6 (4.7–6.5) | 19 | 5.7 (4.9–6.6) | ns | 24 | 6.8 (5.6–7.9) | 30 | 7.8 (6.9–8.8) | 19 | 7.9 (6.9–8.9) | ns |
|  | M | 20 | 6.5 (5.5–7.5) | 21 | 5.8 (4.9–6.6) | 12 | 6.1 (5.1–7.1) | ns | 20 | 7.2 (6.1–8.3)^a^ | 21 | 8.1 (7.3–8.8)^b^ | 12 | 8.5 (8.4–8.7)^b^ | **0.003** |
| **MPM (pg)** | F | 24 | 2.4 (2.1–2.6) | 30 | 2.3 (2.5–2.5) | 19 | 2.2 (2.1–2.4) | ns | 24 | 2.2 (1.9–2.4) | 30 | 2.2 (2.1–2.4) | 19 | 2.2 (2.1–2.4 | ns |
|  | M | 20 | 2.3 (2.1–2.6) | 21 | 2.2 (2.1–2.3) | 12 | 2.5 (2.2–2.8) | ns | 20 | 2.1 (1.9–2.3) | 21 | 2.2 (2.1–2.3) | 12 | 2.3 (2.2–2.5) | ns |
| **PMDW (pg)** | F | 24 | 0.9 (0.8–1.0) | 30 | 0.9 (0.8–0.9) | 19 | 0.9 (0.8–0.9) | ns | 24 | 0.8 (0.6–0.9)^a^ | 30 | 0.9 (0.8–0.9)^b^ | 19 | 0.9 (0.8–0.9)^b^ | **0.001** |
|  | M | 20 | 0.9 (0.8–1.0)^a^ | 21 | 0.9 (0.9–0.9)^a^ | 12 | 1.0 (0.9–1.2)^b^ | **0.029** | 20 | 0.8 (0.6–0.9) | 21 | 0.8 (0.7–0.8) | 12 | 0.8 (0.8–0.9) | ns |
| **AGREG (n)** | F | 24 | 269.1 (29.1–509.2) | 30 | 163.7 (26.3–301.1) | 19 | 299.3 (63.5–534.9) | ns | 24 | 36.6 (1.5–71.7) | 30 | 29.7 (10.7–48.7) | 19 | 37.0 (19.5–54.5) | **0.004** |
|  | M | 20 | 109.8 (3.7–215.9) | 21 | 88.5 (3.6–173.3) | 12 | 189.3 (46.2–332.3) | ns | 20 | 66.7 (25.3–158.6) | 21 | 21.6 (15.3–27.9) | 12 | 69.3 (5.5–133.1) | ns |

**Supplementary Table 2**. Platelet variables in whole blood (WB) and platelet-rich plasma (PRP) according to age group and hematological enrichment tier. Values are presented as median (25th–75th percentile). Kruskal–Wallis tests followed by Dunn’s post hoc tests were used for comparisons among age groups and enrichment tiers. P-values were adjusted using the Benjamini–Hochberg procedure. Different superscript letters indicate significant differences among enrichment tiers within age group, and asterisks indicate significant differences between age groups within the same tier (adjusted p < 0.05).

Abbreviations: PLT, platelet count; PCT, plateletcrit; MPV, mean platelet volume; LARGEPLT, large platelets; MPC, mean platelet protein content; PDW, platelet distribution width; PCDW, platelet protein distribution width; MPM, mean platelet mass; PMDW, platelet mass distribution width; AGREG, platelet aggregates.

|  |  | **WB** | | | | | |  | **PRP** | | | | | |  |
| --- | --- | --- | --- | --- | --- | --- | --- | --- | --- | --- | --- | --- | --- | --- | --- |
|  | **Age (years)** | **N** | **Q1 (< 1.5)** | **N** | **Q2 (1.5-2.5)** | **N** | **Q3 (> 2.5)** | **p-value** | **N** | **Q1 (< 1.5)** | **N** | **Q2 (1.5-2.5)** | **N** | **Q3 (> 2.5)** | **p-value** |
| **PLT (10^3^/mL)** | 1-4 | 18 | 270.5 (221.2-319.9) | 24 | 314.7 (263.7-365.7) | 16 | 253.8 (176.3-331.4) | ns | 18 | 277.1 (168.2-386.0) ^a^ | 24 | 650.0 (532.4-767.5) ^b^ | 16 | 849.8 (596.7-1102.8) ^b^ | **< 0.001** |
|  | 5-9 | 13 | 234.1 (189.9-278.2) | 14 | 326.6 (267.7-385.5) | 9 | 261.4 (193.4-329.4) | ns | 13 | 204.6 (109.9-299.2) ^a^ | 14 | 636.8 (508.7-764.9) ^b^ | 9 | 765.2 (587.9-942.5) ^b^ | **< 0.001** |
|  | 10-15 | 12 | 307.7 (213.4-401.9) | 13 | 342.8 (266.4-419.3) | 5 | 369.0 (285.6-452.4) | ns | 12 | 265.4 (120.9-409.9) ^a^ | 13 | 662.1 (481.7-842.4) ^b^ | 5 | 1159.0 (932.2-1385.7) ^b^ | **< 0.001** |
| **PCT (%)** | 1-4 | 18 | 0.30 (0.2-0.3) | 24 | 0.3 (0.2-0.4) | 16 | 0.3 (0.1-0.3) | ns | 18 | 0.3 (0.1-0.4) ^a^ | 24 | 0.8 (0.6-0.9) ^b^ | 16 | 1.2 (0.7-1.6) ^b^ | **< 0.001** |
|  | 5-9 | 13 | 0.28 (0.2-0.3) | 14 | 0.3 (0.2-0.3) | 9 | 0.3 (0.2-0.3) | ns | 13 | 0.3 (0.1-0.4) ^a^ | 14 | 0.8 (0.6-1.1) ^b^ | 9 | 1.1 (0.9-1.3) ^b^ | **< 0.001** |
|  | 10-15 | 12 | 0.37 (0.2-0.5) | 13 | 0.3 (0.2-0.5) | 5 | 0.3 (0.2-0.4) | ns | 12 | 0.3 (0.1-0.5) ^a^ | 13 | 1.0 (0.7-1.3) ^b^ | 5 | 1.6 (1.4-1.9) ^b^ | **< 0.001** |
| **MPV (fL)** | 1-4 | 18 | 11.4 (9.7-13.1) | 24 | 10.8 (9.8-11.8) | 16 | 12.3 (8.3-16.3) | ns | 18 | 11.1 (9.6-12.5) ^a^ | 24 | 12.5 (10.7-14.2) ^a*^ | 16 | 13.8 (12.1-15.4) ^b^ | **< 0.001** |
|  | 5-9 | 13 | 12.6 (9.2-16.1) | 14 | 10.1 (9.2-10.9) | 9 | 12.5 (9.3-15.8) | ns | 13 | 12.3 (10.1-14.4) | 14 | 13.3 (11.8-14.7) | 9 | 15.4 (14.3-16.5) | ns |
|  | 10-15 | 12 | 12.7 (9.7-15.6) | 13 | 11.4 (9.4-13.4) | 5 | 10.0 (9.8-10.3) | ns | 12 | 12.2 (10.1-14.3) ^a^ | 13 | 15.8 (14.8-16.7) ^b^ | 5 | 15.0 (13.3-16.6) ^a^ | **< 0.001** |
| **LARGEPLT (10^3^/mL)** | 1-4 | 18 | 21.8 (6.0-37. 6) | 24 | 18.8 (9.1-28.6) | 16 | 17.4 (11.1-23.8) | ns | 18 | 29.4 (6.7-52.1) ^a^ | 24 | 84.1(47.2-120.8)^b*^ | 16 | 160.1 (79.4-240.8) ^b^ | **< 0.001** |
|  | 5-9 | 13 | 24.1 (9.5-38.6) | 14 | 13.7 (8.1-19.5) | 9 | 23.3 (12.7-33.8) | ns | 13 | 39.3 (6.8-71.7) ^a^ | 14 | 107.5 (63.8-151.3) ^b*^ | 9 | 177.2 (139.3-215.1) ^b^ | **< 0.001** |
|  | 10-15 | 12 | 32.9 (6.8-58.9) | 13 | 27.7 (3.5-51.9) | 5 | 13.3 (10.2-16.4) | ns | 12 | 33.6 (4.4-62.7) ^a^ | 13 | 172.9 (128.2-217.6) ^b*^ | 5 | 245.5 (175.6-315.3) ^b^ | **< 0.001** |
| **MPC (g/dL)** | 1-4 | 18 | 23.6 (22.5-24.7) | 24 | 23.7 (22.9-24.4) | 16 | 22.8 (20.8-24.7) | ns | 18 | 22.9 (20.7-25.1) ^a*^ | 24 | 21.5 (19.4-23.4) ^a*^ | 16 | 20.7 (18.8-22.5) ^a^ | **< 0.001** |
|  | 5-9 | 13 | 22.6 (20.9-23.9) ^a^ | 14 | 24.6 (23.7-25.5) ^b^ | 9 | 22.9 (21.2-24.5) ^a^ | **0.029** | 13 | 20.0 (18.1-21.8) ^a^ | 14 | 20.6 (18.9-22.3) ^b^ | 9 | 18.2 (16.8-19.5) ^a^ | **< 0.001** |
|  | 10-15 | 12 | 22.4 (20.9-23.9) | 13 | 23.9 (22.8-25.1) | 5 | 23.7 (22.9-24.6) | ns | 12 | 21.5 (18.8-24.1) ^a^ | 13 | 17.8 (17.1-18.6) ^b*^ | 5 | 18.5 (16.8-20.2) ^a^ | **0.026** |
| **PDW (%)** | 1-4 | 18 | 65.1 (57.9-72.3) | 24 | 64.3 (57.8-70.8) | 16 | 65.4 (59.4-71.4) | ns | 18 | 56.5 (48.8-64.3) | 24 | 56.9 (53.1-60.8) | 16 | 59.7 (55.8-63.6) | ns |
|  | 5-9 | 13 | 66.6 (60.2-73.1) | 14 | 58.7 (55.0-62.4) | 9 | 65.9 (59.2-72.5) | ns | 13 | 55.9 (52.3-59.5) | 14 | 57.6 (55.7-59.6) | 9 | 58.8 (57.7-60.0) | ns |
|  | 10-15 | 12 | 63.3 (58.1-68.5) | 13 | 65.3 (58.1-72.5) | 5 | 57.1 (53.9-60.3) | ns | 12 | 59.3 (51.1-67.4) | 13 | 58.4 (56.9-59.9) | 5 | 56.9 (56.0-57.9) | ns |
| **PCDW (g/dL)** | 1-4 | 18 | 5.9 (5.1-6.8) | 24 | 5.8 (5.1-6.5) | 16 | 5.4 (4.6-6.2) | ns | 18 | 6.4 (5.3-7.6) ^a*^ | 24 | 7.5 (6.5-8.5) ^b^ | 16 | 7.7 (6.6-8.8) ^b^ | **< 0.001** |
|  | 5-9 | 13 | 6.1 (5.2-7.1) | 14 | 5.1 (4.1-6.1) | 9 | 6.5 (5.5-7.6) | ns | 13 | 7.8 (7.2-8.5) | 14 | 7.9 (7.1-8.7) | 9 | 8.5 (8.3-8.6) | ns |
|  | 10-15 | 12 | 6.2 (5.2-7.3) | 13 | 5.9 (5.1-6.7) | 5 | 6.0 (5.5-6.4) | ns | 12 | 6.7 (5.4-8.0) ^a^ | 13 | 8.6 (8.4-8.8) ^b^ | 5 | 8.4 (8.1-8.7) ^a^ | **< 0.001** |
| **MPM (pg)** | 1-4 | 18 | 2.3 (2.1-2.5) | 24 | 2.2 (2.1-2.3) | 16 | 2.3 (2.1-2.5) | ns | 18 | 2.1 (2.0-2.3) | 24 | 2.2 (2.1-2.3) | 16 | 2.3 (2.1-2.5) | ns |
|  | 5-9 | 13 | 2.3 (2.1-2.5) | 14 | 2.3 (2.1-2.4) | 9 | 2.4 (2.1-2.6) | ns | 13 | 2.0 (1.8-2.1) ^a^ | 14 | 2.2 (2.1-2.3) ^b^ | 9 | 2.2 (2.1-2.3) ^a^ | **0.024** |
|  | 10-15 | 12 | 2.3 (2.0-2.7) | 13 | 2.3 (2.1-2.5) | 5 | 2.1 (2.1-2.2) | ns | 12 | 2.1 (1.8-2.4) | 13 | 2.2 (2.0-2.4) | 5 | 2.2 (2.1-2.3) | ns |
| **PMDW (pg)** | 1-4 | 18 | 0.9 (0.8-0.9) | 24 | 0.9 (0.8-0.9) | 16 | 0.9 (0.8-1.1) | ns | 18 | 0.7 (0.6-0.9) | 24 | 0.7 (0.6-0.8) | 16 | 0.8 (0.7-0.9) | ns |
|  | 5-9 | 13 | 0.9 (0.8-1.1) | 14 | 0.8 (0.8-0.8) | 9 | 0.9 (0.8-1.1) | ns | 13 | 0.6 (0.5-0.7) | 14 | 0.7 (0.7-0.8) | 9 | 0.7 (0.7-0.7) | ns |
|  | 10-15 | 12 | 0.9 (0.7-1.1) | 13 | 0.9 (0.8-1.0) | 5 | 0.8 (0.8-0.9) | ns | 12 | 0.8 (0.6-0.9) | 13 | 0.7 (0.6-0.8) | 5 | 0.7 (0.6-0.8) | ns |
| **AGREG (n)** | 1-4 | 18 | 165.9 (113.1-344.9) | 24 | 123.1 (27.4-218.8) | 16 | 262.6 (67.5-457.8) | ns | 18 | 37.5 (-0.2-75.2) | 24 | 26.5 (9.1-43.9) | 16 | 63.1 (7.2-119.1) | ns |
|  | 5-9 | 13 | 143.3 (7.1-279.6) | 14 | 69.0 (17.9-120.1) | 9 | 185.1(16.2-354.0) | ns | 13 | 71.2 (38.1-180.5) | 14 | 21.0 (14.0-27.9) | 9 | 32.4 (14.2-50.6) | ns |
|  | 10-15 | 12 | 313.1 (60.8-565.4) | 13 | 219.3 (36.6-401.9) | 5 | 348.0 (57.5-638.5) | ns | 12 | 41.7 (9.3-74.1) | 13 | 32.0 (14.7-49.2) | 5 | 38.6 (22.4-54.8) | ns |

**Supplementary Table 3.** Platelet variables in whole blood (WB) and platelet-rich plasma (PRP) according to body condition score (BCS) category and hematological enrichment tier. Values are presented as median (25th–75th percentile). Mann–Whitney U and Kruskal–Wallis/Dunn tests were used, with Benjamini–Hochberg adjustment for multiple comparisons. Different superscript letters indicate significant differences among enrichment tiers within each BCS category, and asterisks indicate significant differences between BCS categories within the same tier (adjusted p < 0.05).

Abbreviations: PLT, platelet count; PCT, plateletcrit; MPV, mean platelet volume; LARGEPLT, large platelets; MPC, mean platelet protein content; PDW, platelet distribution width; PCDW, platelet protein distribution width; MPM, mean platelet mass; PMDW, platelet mass distribution width; AGREG, platelet aggregates.

|  |  | **WB** | | | | | |  | **PRP** | | | | | |  |
| --- | --- | --- | --- | --- | --- | --- | --- | --- | --- | --- | --- | --- | --- | --- | --- |
|  | **BSC** | **N** | **Q1 (< 1.5)** | **N** | **Q2 (1.5-2.5)** | **N** | **Q3 (> 2.5)** | **p-value** | **N** | **Q1 (< 1.5)** | **N** | **Q2 (1.5-2.5)** | **N** | **Q3 (> 2.5)** | **p-value** |
| **PLT (10^3^/mL)** | Underweight | 10 | 259.7 (202.2-317.2) | 10 | 323.7 (283.2-364.2) | 5 | 304.0 (268.4-339.6) | ns | 10 | 222.5 (130.9-314.0) ^a^ | 10 | 627.8 (554.6-700.9) ^b^ | 5 | 798.8 (691.9-905.6) ^b^ | **< 0.001** |
|  | Ideal | 26 | 262.9 (215.7-310.2) | 23 | 326.1 (258.5-393.8) | 16 | 297.9 (212.1-382.6) | ns | 26 | 260.5 (156.1-364.8) ^a^ | 23 | 665.2 (522.01808.3) ^b^ | 16 | 937.2 (634.7-1239.7) ^b^ | **< 0.001** |
|  | Overweight | 8 | 310 (193.5-426.5) | 18 | 324.8 (264.0-385.5) | 10 | 235.1 (151.4-318.8) | ns | 8 | 262.8 (82.9-442.6) ^a^ | 18 | 641.4 (484.4-798.5) ^b^ | 10 | 844.9 (668.8-1021.0) ^b^ | **< 0.001** |
| **PCT (%)** | Underweight | 10 | 0.3 (0.2-0.4) | 10 | 0.3 (0.2-0.4) | 5 | 0.3 (0.3-0.4) | ns | 10 | 0.2 (0.1-0.3) ^a^ | 10 | 0.8 (0.7-0.9) ^b^ | 5 | 1.3 (1.1-1.5) ^c^ | **< 0.001** |
|  | Ideal | 26 | 0.3 (0.26-0.4) | 23 | 0.3 (0.3-0.4) | 16 | 0.3 (0.36-0.4) | ns | 26 | 0. 3 (0.2-0.5) ^a^ | 23 | 0.7 (0.7-1.1) ^b^ | 16 | 1.3 (0.8-1.7) ^b^ | **< 0.001** |
|  | Overweight | 8 | 0.4 (0.3-0.6) | 18 | 0.4 (0.3-0.5) | 10 | 0.2 (0.1-0.3) | ns | 8 | 0.6 (0.1-0.6) ^a^ | 18 | 0.9 (0.7-1.8) ^b^ | 10 | 1.3 (0.9-1.6) ^b^ | **< 0.001** |
| **MPV (fL)** | Underweight | 10 | 12.5 (9.5-15.5) | 10 | 10.6 (9.2-12.1) | 5 | 10.6 (10.1-11.2) | ns | 10 | 9.9 (8.8-11.1) ^a*^ | 10 | 13.0 (11.2-14.8) ^b^ | 5 | 16.4 (16.1-16.8) ^c^ | **< 0.001** |
|  | Ideal | 26 | 11.5 (9.3-13.7) | 23 | 10.6 (9.8-11.3) | 16 | 13.2 (8.6-17.7) | ns | 26 | 12.1 (10.1-14.0) | 23 | 13.0 (11.3-14.8) | 16 | 13.9 (12.6-15.3) | ns |
|  | Overweight | 8 | 13.9 (10.7-17.3) | 18 | 11.1 (9.4-12.9) | 10 | 10.8 (10.1-11.4) | ns | 8 | 13.2 (11.6-14.9) | 18 | 14.6 (13.1-16.6) | 10 | 14.5 (12.5-16.4) | ns |
| **LARGEPLT (10^3^/mL)** | Underweight | 10 | 24.7 (8.3-41.1) | 10 | 19.1 (4.7-33.5) | 5 | 19.4 (13.7-25.2) | ns | 10 | 12.7 (0.3-25.1) ^a^ | 10 | 101.8 (57.4-146.2) ^b^ | 5 | 225.6 (195.0-256.2) ^c^ | **< 0.001** |
|  | Ideal | 26 | 20. 5 (6.6-34.4) | 23 | 16.6 (11.3-21.9) | 16 | 20.5 (12.2-28.8) | ns | 26 | 39.5 (10.5-68.7) ^a^ | 23 | 98.9 (55.3-142.5) ^b^ | 16 | 162.3 (89.7-234.8) ^b^ | **< 0.001** |
|  | Overweight | 8 | 44.1 (13.6-74.6) | 18 | 24.1 (3.0-45.2) | 10 | 14.4 (7.4-21.4) | ns | 8 | 40.6 (9.0-72.2) ^a^ | 18 | 135.7 (85.2-186.1) ^b^ | 10 | 190.6 (110.3-270.9) ^b^ | **< 0.001** |
| **MPC (g/dL)** | Underweight | 10 | 23.0 (21.1-24.9) | 10 | 24.1 (23.2-24.9) | 5 | 23.9 (23.3-24.6) | ns | 10 | 24.3 (22.6-26.1) ^a*^ | 10 | 20.5 (18.3-22.8) ^a^ | 5 | 17.3 (16.2-18.5) ^b*^ | **< 0.001** |
|  | Ideal | 26 | 23.4 (22.3-24.6) | 23 | 24.0 (23.2-24.8) | 16 | 22.3 (20.2-24.4) | ns | 26 | 21.2 (18.9-23.5) | 23 | 20.9 (18.8-22.9) | 16 | 20.7 (18.8-22.6) | ns |
|  | Overweight | 8 | 21.5 (20.2-22.8) ^a^ | 18 | 24.0 (22.9-25.2) ^b^ | 10 | 23.8 (22.7-24.9) ^a^ | **0.003** | 8 | 19.8 (17.8-21.9) ^a^ | 18 | 19.5 (18.0-20.9) ^a^ | 10 | 18.9 (17.4-20.3) ^b^ | **< 0.001** |
| **PDW (%)** | Underweight | 10 | 65.2 (56.7-73.8) | 10 | 64.6 (55.9-73.2) | 5 | 68.5 (62.2-74.8) | ns | 10 | 54.1 (48.8-59.4) | 10 | 55.9 (51.3-60.6) | 5 | 59.9 (59.1-60.8) | ns |
|  | Ideal | 26 | 64.8 (59.1-70.5) | 23 | 62.8 (56.9-68.8) | 16 | 61.2 (55.3-67.0) | ns | 26 | 57.4 (49.7-65.1) | 23 | 57.1 (54.3-59.9) | 16 | 60.1 (56.8-63.4) | ns |
|  | Overweight | 8 | 65.7 (59.5-71.6) | 18 | 62.5 (57.2-67.8) | 10 | 66.2 (60.3-72.1) | ns | 8 | 60.4 (54.9-65.8) | 18 | 58.9 (57.7-60.2) | 10 | 56.6 (53.9-59.2) | ns |
| **PCDW (g/dL)** | Underweight | 10 | 6.2 (5.2-7.3) | 10 | 5.9 (4.9-6.8) | 5 | 6.1 (5.2-6.7) | ns | 10 | 6.4 (5.2-7.5) ^a^ | 10 | 7.8 (6.9-8.7) ^a^ | 5 | 8.5 (8.3-8.7) ^b^ | **< 0.001** |
|  | Ideal | 26 | 5.6 (5.0-6.7) | 23 | 5.5 (4.8-6.3) | 16 | 5.7 (4.8-6.6) | ns | 26 | 7.1 (5.9-8.2) | 23 | 7.6 (6.6-8.6) | 16 | 7.9 (6.9-8.9) | ns |
|  | Overweight | 8 | 6.8 (5.8-7.8) | 18 | 5.7 (4.8-6.6) | 10 | 6.1 (5.1-6.9) | ns | 8 | 7.4 (6.2-8.6) ^a^ | 18 | 8.4 (7.9-8.9) ^b^ | 10 | 8.2 (7.6-8.9) ^a^ | **< 0.001** |
| **MPM (pg)** | Underweight | 10 | 2.2 (2.1-2.39) | 10 | 2.2 (2.1-2.4) | 5 | 2.2 (2.1-2.4) | ns | 10 | 2.2 (1.9-2.3) | 10 | 2.2 (2.0-2.3) | 5 | 2.2 (2.1-2.4) | ns |
|  | Ideal | 26 | 2.3 (2.1-2.5) | 23 | 2.3 (2.1-2.4) | 16 | 2.4 (2.1-2.7) | ns | 26 | 2.1 (1.9-2.3) | 23 | 2.2 (2.1-2.4) | 16 | 2.3 (2.2-2.5) | ns |
|  | Overweight | 8 | 2.4 (2.0-2.8) | 18 | 2.33 (2.2-2.5) | 10 | 2.32 (2.1-2.4) | ns | 8 | 2.1 (1.8-2.5) | 18 | 2.3 (2.1-2.4) | 10 | 2.2 (2.1-2.1) | ns |
| **PMDW (pg)** | Underweight | 10 | 0.9 (0.8-1.0) | 10 | 0.9 (0.8-0.9) | 5 | 0.9 (0.9-1.0) | ns | 10 | 0.8 (0.7-0.87) | 10 | 0.7 (0.6-0.8) | 5 | 0.8 (0.78-0.9) | ns |
|  | Ideal | 26 | 0.9 (0.8-1.1) | 23 | 0.9 (0.8-0.9) | 16 | 0.9 (0.8-1.0) | ns | 26 | 0.7 (0.62-0.9) | 23 | 0.8 (0.7-0.8) | 16 | 0.8 (0.7-0.9) | ns |
|  | Overweight | 8 | 0.9 (0.8-1.1) | 18 | 0.9 (0.8-0.9) | 10 | 0.9 (0.9-0.9) | ns | 8 | 0.8 (0.6-0.9) | 18 | 0.8 (0.7-0.9) | 10 | 0.8 (0.7-0.8) | ns |
| **AGREG (n)** | Underweight | 10 | 106.5 (19.9-193.0) ^a*^ | 10 | 84.2 (21.3-147.1) ^a^ | 5 | 137 (24.8-249.2) ^a^ | **0.049** | 10 | 90.8 (30.8-212.4) | 10 | 19.3 (15.2-23.4) | 5 | 26.2 (16.9-35.4) | ns |
|  | Ideal | 26 | 156.9 (12.8-325.9) | 23 | 143.8 (48.9-238.6) | 16 | 268.6 (51.6-485.5) | ns | 26 | 36.59 (10.5-73.6) | 23 | 27.0 (8.99-45.2) | 16 | 62.6 (16.4-118.7) | ns |
|  | Overweight | 8 | 472.4 (199.4-745.3) ^a^ | 18 | 145.8 (20.4-311.9) ^b^ | 10 | 297.5 (69.4-525.6) ^a^ | **0.002** | 8 | 35.8 (5.1-66.5) | 18 | 29.6 (14.5-44.7) | 10 | 40.3 (21.1-59.6) | ns |

**Supplementary Table 4**. Platelet variables in whole blood (WB) and platelet-rich plasma (PRP) according to fertility status and hematological enrichment tier. Values are presented as median (25th–75th percentile). Mann–Whitney U and Kruskal–Wallis/Dunn tests were used, with Benjamini–Hochberg adjustment for multiple comparisons. Different superscript letters indicate significant differences among enrichment tiers within fertility status, and asterisks indicate significant differences between fertility categories within the same tier (adjusted p < 0.05).

Abbreviations: PLT, platelet count; PCT, plateletcrit; MPV, mean platelet volume; LARGEPLT, large platelets; MPC, mean platelet protein content; PDW, platelet distribution width; PCDW, platelet protein distribution width; MPM, mean platelet mass; PMDW, platelet mass distribution width; AGREG, platelet aggregates.

|  |  | **WB** | | | | | |  | **PRP** | | | | | |  |
| --- | --- | --- | --- | --- | --- | --- | --- | --- | --- | --- | --- | --- | --- | --- | --- |
|  | **Fertility** | **N** | **Q1 (< 1.5)** | **N** | **Q2 (1.5-2.5)** | **N** | **Q3 (> 2.5)** | **p-value** | **N** | **Q1 (< 1.5)** | **N** | **Q2 (1.5-2.5)** | **N** | **Q3 (> 2.5)** | **p-value** |
| **PLT (10^3^/mL)** | F | 28 | 265.4 (220.5-310.4) ^a^ | 37 | 322.9 (273.2-372.7) ^b^ | 26 | 284.0 (200.1-368.0) ^a^ | **0.017** | 28 | 248.1 (143.4-352.8) ^a^ | 37 | 652.0 (526.7-777.3) ^b^ | 26 | 899.0 (652.1-1146.0) ^c^ | **< 0.001** |
|  | I | 16 | 280.0 (182.5-377.5) | 14 | 336.1 (251.1-421.1) | 5 | 248.8 (195.9-301.6) | ns | 16 | 255.6 (116.4-394.8) ^a^ | 14 | 660.7 (494.7-826.7) ^b^ | 5 | 812.6 (587.4-1037.8) ^b^ | **< 0.001** |
| **PCT (%)** | F | 28 | 0.3 (0,2-0,4) | 37 | 0.3 (0,3-0,4) | 26 | 0.3 (0,3-0,4) | ns | 28 | 0.3 (0.15-0.5) ^a^ | 37 | 0.9 (0.7-1.1) ^b^ | 26 | 1.3 (0.9-1.7) ^c^ | **< 0.001** |
|  | I | 16 | 0.3 (0,2-0,5) | 14 | 0.4 (0,2-0,5) | 5 | 0.3 (0,2-0,3) | ns | 16 | 0.31 (0.12-0.5) ^a^ | 14 | 0.9 (0.8-1.28) ^b^ | 5 | 1.3 (0.95-1.6) ^b^ | **< 0.001** |
| **MPV (fL)** | F | 28 | 11.3 (9.8-12.9) | 37 | 10.6 (9.7-11.57) | 26 | 11.8 (8.7-14.9) | ns | 28 | 11.5 (9.6-13.5) ^a^ | 37 | 12.9 (11.2-14.8) ^a^ | 26 | 14.2 (12.6-15.8) ^b^ | **< 0.001** |
|  | I | 16 | 13.8 (9.9-17.6) | 14 | 11.4 (9.3-13.4) | 5 | 12.9 (8.2-17.5) | ns | 16 | 12.1 (10.3-13.9) ^a^ | 14 | 15.1 (13.9-16.3) ^b^ | 5 | 16.3 (15.4-17.2) ^b^ | **< 0.001** |
| **LARGEPLT (10^3^/mL)** | F | 28 | 20.9 (6.7-35.1) | 37 | 17.8 (9.1-26.5) | 26 | 18.2 (11.8-24.5) | ns | 28 | 33.3 (4.4-62.1) ^a^ | 37 | 100.4 (52.0-148.7) ^b^ | 26 | 175.4 (101.4-249.3) ^c^ | **< 0.001** |
|  | I | 16 | 34.3 (8.5-60) | 14 | 26.2 (1.7-50.7) | 5 | 19.4 (5.9-32.9) | ns | 16 | 32.3 (6.5-57.9) ^a^ | 14 | 145.1 (110.8-179.3) ^b^ | 5 | 214.2 (168.4-260) ^b^ | **< 0.001** |
| **MPC (g/dL)** | F | 28 | 23.6 (22.7-24.5) ^a^ | 37 | 23.9 (23.2-24.8) ^a^ | 26 | 23.1 (21.4-24.7) ^a^ | **0.001** | 28 | 22.3 (19.9-24.7) ^a*^ | 37 | 20.9 (18.8-22.9) ^a^ | 26 | 20.1 (18.3-21.9) ^a*^ | **< 0.001** |
|  | I | 16 | 21.9 (20.1-23.8) ^a^ | 14 | 24.0 (22.9-25.2) ^b^ | 5 | 22.9 (20.9-24.9) ^a^ | **0.001** | 16 | 20.9 (18.7-23.1) ^a^ | 14 | 18.8 (17.9-19.7) ^a^ | 5 | 16.9 (16.5-17.2) ^b^ | **< 0.001** |
| **PDW (%)** | F | 28 | 63.7 (57.9-69.6) | 37 | 63.6 (56.7-70.5) | 26 | 64.3 (57.8-70.7) | ns | 28 | 56.7 (48.7-64.7) | 37 | 57.2 (53.9-60.6) | 26 | 59.2 (55.9-62.33) | ns |
|  | I | 16 | 66.3 (59.1-73.5) | 14 | 61.9 (57.7-66.2) | 5 | 62.5 (58.7-66.3) | ns | 16 | 58.1 (53.1-63.1) ^a^ | 14 | 58.3 (56.9-59.7) ^a^ | 5 | 58.0 (56.8-59.2) ^a^ | **< 0.001** |
| **PCDW (g/dL)** | F | 28 | 5.8 (4.9-6.6) | 37 | 5.6 (4.73-6.5) | 26 | 5.7 (4.8-6.5) | ns | 28 | 6.7 (5.5-7.9) ^a^ | 37 | 7.7 (6.7-8.6) ^a^ | 26 | 8.0 (7.1-9.2) ^b^ | **< 0.001** |
|  | I | 16 | 6.6 (5.5-7.6) | 14 | 5.9 (5.2-6.7) | 5 | 6.9 (6.2-7.8) | ns | 16 | 7.4 (6.4-8.5) | 14 | 8.6 (8.4-8.8) | 5 | 8.5 (8.4-8.6) | ns |
| **MPM (pg)** | F | 28 | 2.3 (2.2-2.5) | 37 | 2.3 (2.1-2.4) | 26 | 2.3 (2.1-2.5) | ns | 28 | 2.2 (1.9-2.3) | 37 | 2.2 (2.1-2.3) | 26 | 2.3 (2.1-2.5) | ns |
|  | I | 16 | 2.4 (1.9-2.7) | 14 | 2.4 (2.2-2.5) | 5 | 2.3 (2.0-2.6) | ns | 16 | 2.1 (1.8-2.3) | 14 | 2.3 (2.1-2.5) | 5 | 2.2 (2.02-2.3) | ns |
| **PMDW (pg)** | F | 28 | 0.9 (0.9-0.9) | 37 | 0.9 (0.8-0.9) | 26 | 0.9 (0.8-1.0) | ns | 28 | 0.5 (0.7-0.9) | 37 | 0.8 (0.7-0.8) | 26 | 0.8 (0.78-0.9) | ns |
|  | I | 16 | 0.9 (0.8-1.1) | 14 | 0.9 (0.96-1.0) | 5 | 0.9 (0.8-1.02) | ns | 16 | 0.8 (0.6-0.9) | 14 | 0.8 (0.7-0.9) | 5 | 0.7 (0.7-0.8) | ns |
| **AGREG (n)** | F | 28 | 144.6 (16.1-305.3) | 37 | 127.5 (31.1-223.8) | 26 | 211.1 (34.7-387.4) | ns | 28 | 34.3 (11.3-69.8) ^a^ | 37 | 24.6 (10.0-39.3) ^a^ | 26 | 53.7 (8.2-99.2) ^b*^ | **< 0.001** |
|  | I | 16 | 291.5 (48.8-534.1) | 14 | 151.0 (26.7-328.7) | 5 | 493.8 (203.9-783.6) | ns | 16 | 78.9 (21.8-179.6) | 14 | 31.6 (14.2-49.1) | 5 | 27.8 (14.5-41.1) | ns |

**Supplementary Table 5.** Platelet variables in whole blood (WB) and platelet-rich plasma (PRP) according to activity level and hematological enrichment tier. Values are presented as median (25th–75th percentile). Mann–Whitney U and Kruskal–Wallis/Dunn tests were used, with Benjamini–Hochberg adjustment for multiple comparisons. Different superscript letters indicate significant differences among enrichment tiers within activity category, and asterisks indicate significant differences between activity categories within the same tier (adjusted p < 0.05).

Abbreviations: PLT, platelet count; PCT, plateletcrit; MPV, mean platelet volume; LARGEPLT, large platelets; MPC, mean platelet protein content; PDW, platelet distribution width; PCDW, platelet protein distribution width; MPM, mean platelet mass; PMDW, platelet mass distribution width; AGREG, platelet aggregates.

|  |  | **WB** | | | | | |  | **PRP** | | | | | |  |
| --- | --- | --- | --- | --- | --- | --- | --- | --- | --- | --- | --- | --- | --- | --- | --- |
|  | **Activity** | **N** | **Q1 (< 1.5)** | **N** | **Q2 (1.5-2.5)** | **N** | **Q3 (> 2.5)** | **p-value** | **N** | **Q1 (< 1.5)** | **N** | **Q2 (1.5-2.5)** | **N** | **Q3 (> 2.5)** | **p-value** |
| **PLT (10^3^/mL)** | Low | 4 | 326.0 (201.5-450.0) | 8 | 299.0 (243.3-336.5) | 4 | 255.5 (141-387.5) | ns | 4 | 200.5 (160.5-247.5) ^a^ | 8 | 542.1 (436-650.8) ^b^ | 4 | 631.3 (428.3-722.5) ^b^ | **< 0.001** |
|  | Medium | 22 | 276.9 (238-287.5) ^a^ | 28 | 341.2 (282.8-411.8) ^b^ | 13 | 296.4 (232-372) ^b^ | **< 0.001** | 22 | 265.6 (106-352.5) ^a^ | 28 | 685.0 (532.3-782.3) ^b^ | 13 | 1023.3 (694-1501) ^c^ | **< 0.001** |
|  | High | 18 | 250.9 (199.8-304.8) | 15 | 309.2 (281-327.5) | 14 | 268.1 (253-308) | ns | 18 | 247.4 (127.8-371) ^a^ | 15 | 640.4 (489.5-687) ^b^ | 14 | 829.3 (779.8-943.8) ^c^ | **< 0.001** |
| **PCT (%)** | Low | 4 | 0.4 (0.3-0.5) | 8 | 0.4 (0.3-0.4) | 4 | 0.3 (0.2-0.4) | ns | 4 | 0.2 (0.2-0.3) ^a^ | 8 | 0.9 (0.8-0.9) ^b^ | 4 | 1.1 (0.7-1.2) ^b^ | **< 0.001** |
|  | Medium | 22 | 0.3 (0.2-0.3) | 28 | 0.4 (0.3-0.4) | 13 | 0.3 (0.2-0.4) | ns | 22 | 0.3 (0.1-0.4) ^a^ | 28 | 0.9 (0.6-1.1) ^b^ | 13 | 1.4 (0.8-1.8) ^b^ | **< 0.001** |
|  | High | 18 | 0.3 (0.2-0.4) | 15 | 0.3 (0.2-0.4) | 14 | 0.3 (0.3-0.34) | ns | 18 | 0.3 (0.1-0.4) ^a^ | 15 | 0.9 (0.61-0.9) ^b^ | 14 | 1.2 (1.2-1.4) ^b^ | **< 0.001** |
| **MPV (fL)** | Low | 4 | 12.9 (10.1-14.9) | 8 | 12.4 (10.2-14.6) | 4 | 16.7 (10.7-16.9) | ns | 4 | 10.8 (9.5-11.8) ^a^ | 8 | 16.1 (15.3-17.0) ^b*^ | 4 | 16.3 (15.5-16.6) ^a^ | **< 0.001** |
|  | Medium | 22 | 11.3 (9.5-11.9) | 28 | 10.3 (9.5-11.3) | 13 | 10.8 (10-11.5) | ns | 22 | 11.9 (10-13.9) | 28 | 13.2 (11.7-14.7) | 13 | 13.3 (11.1-15) | ns |
|  | High | 18 | 13.1 (10.2-13.3) | 15 | 10.8 (10-10.9) | 14 | 11.8 (10.1-12.0) | ns | 18 | 11.8 (9.6-13.7) ^a^ | 15 | 12.9 (10.4-15.5) ^a^ | 14 | 15.2 (14-16.5) ^b^ | **< 0.001** |
| **LARGEPLT (10^3^/mL)** | Low | 4 | 48.0 (9.5-58.5) | 8 | 34.1 (12-30) | 4 | 22.3 (17.5-27.3) | ns | 4 | 16.8 (3.8-19) ^a^ | 8 | 144.1 (113.5-175.5) ^b^ | 4 | 189.3 (103.5 -220.8) b | **< 0.001** |
|  | Medium | 22 | 21.6 (9-28.3) | 28 | 15.4 (11.8-18.3) | 13 | 15.9 (12-24) | ns | 22 | 37.0 (5.5-51) ^a^ | 28 | 106.9 (68.3-140) ^b^ | 13 | 174.2 (79-256) ^c^ | **< 0.001** |
|  | High | 18 | 25.9 (10.3-42.5) | 15 | 20.2 (8-24) | 14 | 19.6 (11.3-19) | ns | 18 | 33.2 (45.3-46.3) ^a^ | 15 | 106.4 (36.5-130.5) ^b^ | 14 | 186.4 (142.5-226.8) ^c^ | **< 0.001** |
| **MPC (g/dL)** | Low | 4 | 22.8 (22-24.2) | 8 | 23.8 (22.9-25.5) | 4 | 20.9 (20.2-24.3) | ns | 4 | 21.8 (18.8-24.9) | 8 | 18.8 (18.2-19.2) | 4 | 189.3 (103.5-220.8) | ns |
|  | Medium | 22 | 23.3 (21.8-24.8) ^a^ | 28 | 24.3 (23.8-24.9) ^a^ | 13 | 23.3 (22.8-24.1) ^b^ | **< 0.001** | 22 | 21.4 (18.5-24.5) ^a^ | 28 | 20.7 (18.8-23.8) ^a^ | 13 | 21.1 (19.4-22.9) ^b^ | **< 0.001** |
|  | High | 18 | 22.9 (22.1-24.5) | 15 | 23.7 (22.9-24.5) | 14 | 23.3 (22.8-24.7) | ns | 18 | 21.9 (18-24.8) | 15 | 20.3 (17.9-23.1) | 14 | 18.8 (17.4-20.1) | ns |
| **PDW (%)** | Low | 4 | 64.8 (59.8-67.4) | 8 | 62.5 (59.5-65.5) | 4 | 61.4 (54.4-63.5) | ns | 4 | 53.9 (51.6-56.5) | 8 | 58.2 (56.9-59.8) | 4 | 60.2 (58.5-60.5) | ns |
|  | Medium | 22 | 64.1 (57.2-70.4) | 28 | 61.6 (58.4-63.85) | 13 | 63.3 (56.5-63.4) | ns | 22 | 58.2 (55.1-59.9) | 28 | 57.5 (56.8-59.7) | 13 | 57.9 (55-60.3) | ns |
|  | High | 18 | 66.3 (59.9-69.2) | 15 | 65.9 (58.1-73.9) | 14 | 65.3 (60.1-71.9) | ns | 18 | 56.7 (51.9-58.8) | 15 | 57.3 (55.9-60.6) | 14 | 59.6 (58.1-60.9) | ns |
| **PCDW (g/dL)** | Low | 4 | 6.3 (5.6-6.4) | 8 | 6.0 (5.25-6.3) | 4 | 6.4 (6.2-6.7) | ns | 4 | 6.9 (6.7-7.5) ^a^ | 8 | 8.8 (8.7-8.9) ^b^ | 4 | 8.5 (8.2-8.8) ^a^ | **< 0.001** |
|  | Medium | 22 | 6.2 (5.1-7.2) | 28 | 5.3 (4.3-6.1) | 13 | 5.4 (4.5-6.2) | ns | 22 | 7.2 (5.6-8.5) | 28 | 7.7 (7-8.6) | 13 | 7.5 (6.7-8.6) | ns |
|  | High | 18 | 6.0 (4.8-6.7) | 15 | 6.1 (5.2-6.7) | 14 | 6.1 (4.7-7.1) | ns | 18 | 6.8 (5.4-8.1) ^a^ | 15 | 7.9 (7-8.8) ^b^ | 14 | 8.6 (8.5-8.7) ^b^ | **< 0.001** |
| **MPM (pg)** | Low | 4 | 2.4 (2.1-2.7) | 8 | 2.5 (2.4-2.7) | 4 | 2.5 (2.3-2.6) | ns | 4 | 1.9 (1.8-2.0) ^a^ | 8 | 2.4 (2.3-2.6) ^b*^ | 4 | 2.3 (2.1-2.3) ^b^ | **< 0.001** |
|  | Medium | 22 | 2.3 (2.1-2.5) | 28 | 2.3 (2.2-2.4) | 13 | 2.3 (2.1-2.4) | ns | 22 | 2.1 (1.9-2.3) | 28 | 2.2 (2.1-2.4) | 13 | 2.3 (2.1-2.5) | ns |
|  | High | 18 | 2.4 (2.2-2.5) | 15 | 2.2 (2.1-2.3) | 14 | 2.3 (2.0-2.5) | ns | 18 | 2.2 (1.9-2.2) | 15 | 2.1 (2.1-2.2) | 14 | 2.2 (2.1-2.4) | ns |
| **PMDW (pg)** | Low | 4 | 0.9 (0.9-1.1) | 8 | 0.9 (0.9-1.1) | 4 | 1.1 (0.9-1.1) | ns | 4 | 0.7 (0.65-0.8) ^a^ | 8 | 0.8 (0.8-0.9) ^a*^ | 4 | 0.8 (0.8-0.88) ^a^ | **< 0.001** |
|  | Medium | 22 | 0.9 (0.8-0.91) | 28 | 0.9 (0.8-0.92) | 13 | 0.9 (0.9-0.92) | ns | 22 | 0.8 (0.6-0.82) | 28 | 0.8 (0.7-0.9) | 13 | 0.8 (0.7-0.9) | ns |
|  | High | 18 | 0.9 (0.88-1.0) | 15 | 0.9 (0.8-0.92) | 14 | 0.9 (0.8-0.93) | ns | 18 | 0.8 (0.6-0.84) ^a^ | 15 | 0.7 (0.68-0.8) ^b^ | 14 | 0.8 (0.79-0.86) ^a^ | **< 0.001** |
| **AGREG (n)** | Low | 4 | 425.3 (75.8-680) | 8 | 182.0 (51.3-95.5) | 4 | 222.3 (64-301.8) | ns | 4 | 38.8 (9.8-41) | 8 | 41.6 (23.5-61.8) | 4 | 34.5 (16.8-45.3) | ns |
|  | Medium | 22 | 126.5 (27.8-131.9) | 28 | 99.4 (30.8-146) | 13 | 284.5 (24-512) | ns | 22 | 31.7 (12.3-26) | 28 | 26.7 (16.8-26.3) | 13 | 60.0 (14-52) | ns |
|  | High | 18 | 240.0 (34.3-297) | 15 | 168.9 (36-294.5) | 14 | 240.7 (30.3-410) | ns | 18 | 17.8 (12.3-53.8) ^a^ | 15 | 17.9 (10.5-24) ^a^ | 14 | 44.1 (18.8-69.3) ^b^ | **< 0.001** |
